# Exploring the association between sleep insufficiency and self-reported cardiovascular disease among northeastern Greeks

**DOI:** 10.5935/1984-0063.20220069

**Published:** 2022

**Authors:** Petros N. Fountoulakis, Aikaterini Terzoudi, Dimitrios Tsiptsios, Andreas S. Triantafyllis, Anestis Matziridis, Eleni Leontidou, Apostolos Manolis, Konstantinos Tsamakis, Andreas Ouranidis, Paschalis Steiropoulos, Theofanis Vorvolakos, Aspasia Serdari, Gregory Tripsianis

**Affiliations:** 1 Asklepeion Hospital, Department of Cardiology - Athens - Attiki - Greece; 2 Medical School, Democritus University of Thrace, Department of Neurology - Alexandroupolis - Thrace - Greece; 3 Medical School, Democritus University of Thrace, Laboratory of Medical Statistics - Alexandroupolis - Thrace - Greece; 4 King’s College London, Institute of Psychiatry, Psychology and Neuroscience - London - London - United Kingdom; 5 Aristotle University of Thessaloniki, School of Chemical Engineering - Thessaloniki - Thessaloniki - Greece; 6 Medical School, Democritus University of Thrace, Department of Psychiatry - Alexandroupolis - Thrace - Greece; 7 Medical School, Democritus University of Thrace, Department of Pneumonology - Alexandroupolis - Thrace - Greece; 8 Medical School, Democritus University of Thrace, Department of Child and Adolescent Psychiatry - Alexandroupolis - Thrace - Greece

**Keywords:** Sleep Insufficiency, Stroke, Cardiovascular Disease, Angina

## Abstract

**Objective:**

To explore the association of sleep characteristics with cardiovascular disease (CVD) using self-reported questionnaires.

**Material and Methods:**

957 adults between 19 and 86 years old were enrolled in this cross-sectional study. The participants were classified into three groups [short (<6h), normal (6-8h), and long (>8h) sleepers] by using multistage stratified cluster sampling. CVD was defined by a positive response to the questions: “Have you been told by a doctor that you have had a heart attack or angina or stroke or have you undergone bypass surgery?”. Sleep quality, utilizing Epworth sleepiness scale, Athens insomnia scale, Pittsburgh sleep quality index and Berlin questionnaire, was also examined.

**Results:**

Prevalence of CVD was 9.5%. Individuals with CVD exhibited reduced sleep duration by 33 min (*p*<0.001) and sleep efficiency by 10% (*p*<0.001). In multivariable logistic regression analysis, adjusting for subjects’ sociodemographic, lifestyle habits and health related characteristics, short sleep duration was almost three times more frequent in patients with CVD (aOR=2.86, *p*<0.001 in the entire sample; aOR=2.68, *p*=0.019 in women and aOR=2.57, *p*=0.009 in men). Furthermore, CVD was significantly associated with excessive daytime sleepiness (aOR=2.02, *p*=0.026), insomnia (aOR=1.93, *p*=0.010), poor sleep quality (aOR=1.90, *p*=0.006) and increased risk of obstructive sleep apnea (aOR=2.08, *p*=0.003).

**Conclusion:**

Our study highlights a strong correlation of sleep insufficiency with CVD and promotes early pharmacological or cognitive behavioral interventions in order to protect cardiovascular health.

## INTRODUCTION

Sleep represents one of the most natural and inseparable life procedures, occupying a third of our everyday time and allowing us to overcome the daily physical and psychological stress^1,2^. The American Academy of Sleep Medicine and the Sleep Research Society recommend a mean period of six to eight hours of sleep per day for preservation of its beneficial health effects^3^. However, the demanding lifestyle of modern society has extended the working schedule favoring sedentary life and stress^4^. As a result, a third of the general population is affected by sleep disturbances, with almost 30% of individuals reporting chronic sleep problems, such as excessive daytime sleepiness and insomnia and consequent deleterious effects on metabolic, immune and endocrine systems^5^.

Cardiovascular disease (CVD) including ischemic heart disease and stroke, represents a major cause of global morbidity and mortality with increasing prevalence^6,7^. Sleep disorders have gradually become an upcoming and modifiable risk factor for CVD, as a growing number of studies points towards a bidirectional relationship of sleep insufficiency with arterial and pulmonary hypertension, diabetes mellitus, coronary artery disease, heart failure, atrial fibrillation, stroke, and overall mortality^8-10^. However, there are conflicting evidence emphasizing shorter (≤6h/day) and less frequently longer (≥8h/day) sleep duration, as being associated with a significant risk of CVD^11,12^.

Our research group utilizing self-reported questionnaires has recently exhibited that sleep pathology is associated with increased prevalence of anxiety^13^, depression^14^, diabetes mellitus^15^, hypertension^16^ and dyslipidemia^17^. In this paper, we aimed to investigate possible correlations between sleep insufficiency and CVD considering several sociodemographic characteristics, lifestyle habits and health related characteristics of the participants.

## MATERIAL AND METHODS

### Study sample and research design

The study population in this cross-sectional study consisted of 957 participants, 439 (45.9%) males and 518 (54.1%) females, with a mean age of 49.62 ± 14.79 years (range, 19-86 years; median age, 50 years). The sample selection was based on a two-stage stratified sampling scheme on all adults living in the region of Thrace and it was conducted between September 2016 and May 2019. Thrace, the Northeastern prefecture of Greece, is characterized by cultural diversity with various national, ethnolinguistic and religious groups. Its population consists of: a.) the indigenous Christian Orthodox population (65% of the region population), b.) the Muslim minority, which is the dominant minority group (30% of the population in Thrace) including the Pomaks and the Roma-Gypsies, and c.) the descendants of Armenian refugees and a lot of expatriated Greeks from countries of the former Soviet Republics who settled in Thrace (estimated 5% of the population in Thrace). In the first stage of the sampling procedure, the area of Thrace was divided in two strata by the degree of urbanization. The urbanization levels were urban (≥10,000 inhabitants) and semi-urban or rural (<10,000 inhabitants) areas. According to the 2011 census, which constituted the sampling frame in our study, the urban population of Thrace accounted for approximately 40% of the total population of this area. In the second stage, subjects were recruited proportionally to each stratum size, through a method of random generation of telephone numbers on the basis of the area code. After the aim of the study was explained to them, the participants agreed to have field researchers visit their home and to complete the questionnaires of the study in an hour-long interview. The overall response rate was 71%. The sampling scheme ensured that the sample was randomly selected and representative of the general population of Thrace.

### Ethics

Informed consent was obtained from all participants of the study. All procedures performed in the study were in accordance with the ethical standards of the Democritus University Ethics Committee and with the standards of the Helsinki declaration (1964) and its later amendments. The study protocol was approved by the Institutional Ethics Committee (Protocol Number 42570/294).

### Covariates

A structured questionnaire was used to collect: a.) standard sociodemographic characteristics (gender, age, place of residence, education level, marital, cultural, employment, and financial status), b.) lifestyle and dietary habits (smoking status, alcohol consumption, daily coffee consumption, caffeine consumption in the evening, adherence to the Mediterranean diet, time watching TV or using a computer before bedtime, physical activity and nap during the day), and c.) health related characteristics (subjective general health status, body mass index, chronic disease morbidity, anxiety, depression, and use of sleep medication) of the participants (Appendix 1).

### Estimation of sleep duration and sleep efficiency

Participants provided information on their nighttime sleep by answering the following sleep questions of the questionnaire: “At what time do you normally go to bed?”, “At what time do you normally get up?” and “On average, how many hours do you sleep per day?” Responses were obtained for an average weekday and weekend day over the previous month. Time in bed was calculated as the difference between bedtime and rise time. As a proxy of the overall time in bed or sleep duration on a weekly basis, weighted mean measures were calculated using the following formulas: weighted time in bed = 5/7*(time in bed on a weekday) + 2/7*(time in bed on a weekend day) and weighted sleep duration = 5/7*(sleep duration on a weekday) + 2/7*(sleep duration on a weekend day). Sleep efficiency refers to the percentage of time a person sleeps in relation to the amount of time a person spends in bed and was calculated as the ratio of sleep duration and time in bed X 100. Participants were then classified into the following three sleep categories according to calculated sleep duration: short (<6 hours), normal (6-8 hours) and long sleepers (>8 hours)^18^.

### Assessment of sleep quality

Sleep quality was assessed with the Greek versions of Epworth Sleepiness Scale (ESS)^19^, Athens insomnia scale (AIS)^20^, Pittsburgh sleep quality index (PSQI)^21^, and Berlin questionnaire (BQ)^22^ that evaluate excessive daytime sleepiness, insomnia, sleep quality and risk of obstructive sleep apnea (OSA), respectively. With regards to insomnia characteristics, participants were asked whether they experienced difficulties initiating or maintaining sleep or early morning awakenings.

### Definition of CVD

CVD was defined by a positive response to the following questions: “Have you been told by a doctor that you have had a heart attack, angina (chest pain or exertion that is relieved by medication) or have you undergone bypass surgery?” or “Have you been told by a doctor that you have had a stroke?”^23^.

### Statistical analysis

Statistical analysis of the data was performed using IBM Statistical Package for the Social Sciences (SPSS), version 19.0 (IBM Corp., Armonk, NY, USA). The normality of quantitative variables was tested with Kolmogorov-Smirnov test. Quantitative variables were expressed as mean ± standard deviation (SD) and qualitative variables were expressed as absolute and relative (%) frequencies. In particular, mean estimated time of sleep characteristics (i.e., bedtime, rise time, time in bed, and sleep duration) were expressed as HH:MM. We conducted the following analyses: (i) in the univariate analysis, the association of cardiovascular diseases with subjects’ characteristics, sleep characteristics and sleep disorders were assessed using the chi-square test and Student’s t-test; (ii) multivariable stepwise logistic regression analysis was used to explore the independent risk factors for cardiovascular diseases, controlling for all subjects’ characteristics; (iii) for the evaluation of the effect of sleep duration and sleep disorders on the prevalence of cardiovascular diseases, two different logistic regression models were constructed: model 1 (crude, unadjusted) and model 2 (adjusted for subjects’ sociodemographic, lifestyle habits and health related characteristics). Odds ratios (OR) with their 95% confidence intervals (CI) were estimated as the measure of the above associations. In all the above mentioned multivariable backward stepwise logistic regression models all sociodemographic characteristics (gender, age, marital status, cultural status, place of residence, education level, working status, financial status), lifestyle habits (smoking status, alcohol consumption, daily coffee consumption, caffeine consumption in the evening, adherence to the Mediterranean diet, time watching TV or using a computer before bedtime, physical activity, nap during the day) and health related characteristics (subjective general health status, BMI, chronic disease morbidity, anxiety, depression and use of sleep medication) were initially entered as potential confounders; in the sequence, variables were discarded at a *p*-value more than 0.20.

Receiver operating characteristic (ROC) analysis was used to provide the ability of sleep duration to classify subjects with cardiovascular diseases. The area under the ROC curve (AUC), sensitivity and specificity were estimated. The optimal cutoff value of the sleep duration that differentiates subjects with cardiovascular diseases from those without cardiovascular diseases was derived according to Youden index^24^. All tests were two tailed and statistical significance was considered for *p*-values<0.05.

## RESULTS

### Participants’ characteristics

Subjects’ sociodemographic, lifestyle and health related features are outlined in Tables 1 and 2. Mean self-reported sleep duration was 6hrs and 19mins on workdays and 6hrs and 45mins on weekends; 31.7% and 22.9% of the participants reported short sleep duration (<6hrs), while 7.9% and 14.2% reported long sleep duration (>8hrs) on workdays and on weekends, respectively. Sleep related medications were regularly used by 6.9% of the participants of our study.

**Table 1:** Prevalence of cardiovascular disease (CVD) in relation to subjects’ demographic characteristics.

		CVD		p-value
	Total sample	Frequency	Proportion (%)	
Gender				0.003
Females	518 (54.1)	36	6.9	
Males	439 (45.9)	55	12.5	
Age (years)				<0.001
≤60	717 (74.9)	12	1.7	
>60	240 (25.1)	79	32.9	
Marital status				<0.001
Married	645 (67.4)	71	11.0	
Single	196 (20.5)	0	0.0	
Divorced	36 (+3.8)	0	0.0	
Widowed	80 (8.4)	20	25.0	
Cultural status				0.772
Greek Christians	632 (66.1)	63	10.0	
Greek Muslims	273 (28.5)	24	8.8	
Expatriated Greeks	52 (5.4)	4	7.7	
Place of residence				<0.001
Urban	416 (43.5)	8	1.9	
Rural	541 (56.5)	83	15.3	
Education level				<0.001
Low	313 (32.7)	59	18.8	
Medium	340 (35.5)	20	5.9	
High	304 (31.8)	12	3.9	
Working Status				0.114
Employed	872 (91.1)	87	10.0	
Unemployed	85 (8.9)	4	4.7	
Financial status (n=812)				<0.001
Low	476 (49.7)	55	11.6	
Medium	200 (20.9)	4	2.0	
High	136 (14.2)	8	5.9	

**Table 2:** Prevalence of cardiovascular disease (CVD) in relation to subjects’ lifestyle habits and health related characteristics.

		CVD		
	**Total sample**	**Frequency**	**Proportion (%)**	**p-value**
Smoking ever				0.040
Never smoked	369 (38.6)	26	7.0	
Current or ex-smoker	588 (61.4)	65	11.1	
Alcohol consumption				0.005
Never	488 (51.0)	59	12.1	
Occasionally or daily	469 (49.0)	32	6.8	
Coffee consumption				0.730
None	84 (8.8)	7	8.3	
1-2 cups/day	564 (58.9)	56	9.9	
3-4 cups/day	260 (27.2)	21	8.1	
>4 cups/day	49 (5.1)	6	12.2	
Caffeine consumption in the evening (>6 p.m.)				0.001
No	415 (43.4)	55	13.3	
Yes	542 (56.6)	36	6.6	
Adherence to MED diet				<0.001
Low	743 (77.6)	83	11.2	
High	214 (22.4)	8	3.7	
Time watching TV or using a computer before bedtime				<0.001
<1 hour	120 (12.5)	4	3.3	
1-2 hours	326 (34.1)	16	4.9	
>2 hours	511 (53.4)	71	13.9	
Physical activity				0.052
Low	805 (84.1)	83	10.3	
High	152 (15.9)	8	5.3	
Nap during the day				0.533
No	721 (75.3)	71	9.8	
Yes	236 (24.7)	20	8.5	
Subjective health status				<0.001
Bad	220 (23.0)	59	26.8	
Good	737 (77.0)	32	4.3	
BMI status				<0.001
Normal	328 (34.3)	12	3.7	
Overweight	272 (28.4)	23	8.5	
Obese	357 (37.3)	56	15.7	
Chronic disease morbidity				<0.001
No	517 (54.0)	0	0.0	
Yes	440 (46.0)	91	20.7	
Anxiety symptoms				0.137
No	635 (66.4)	54	8.5	
Yes	322 (33.6)	37	11.5	
Depression symptoms				<0.001
No	685 (71.6)	36	5.3	
Yes	272 (28.4)	55	20.2	
Use of sleep medication				0.579
No	891 (93.1)	86	9.7	
Yes	66 (6.9)	5	7.6	

### The prevalence of CVD

The prevalence of CVDs was 9.5% (91 subjects; 95%CI=7.8% to 11.5%) and its relation to participants’ characteristics is shown in Tables 1 and 2. Multivariable logistic regression analysis showed that the strongest risk factor for CVD was age older than 60 years (aOR=23.44, *p*<0.001) (Table 3).

**Table 3:** Significant determinants of cardiovascular disease (CVD) obtained by multivariate logistic regression models.

Characteristics	aOR	95%CI	p-value
Age >60 years	23.44	12.08-45.47	<0.001
Low financial status	2.38	1.11-5.06	0.025
Current smoking	2.21	1.27-3.86	0.005
Low adherence to Mediterranean diet	2.19	1.25-3.83	0.006
Watching TV or using a computer before bedtime for more than 2 hours	2.20	1.20-4.05	0.011
Obesity	2.80	1.65-4.78	<0.001
Depression symptoms	2.44	1.42-4.18	0.001

Notes: aOR = Adjusted odds ratio; CI = Confidence interval; All subjects’ sociodemographic characteristics (gender, age, marital status, cultural status, place of residence, education level, working status, financial status), lifestyle habits (smoking status, alcohol consumption, daily coffee consumption, caffeine consumption in the evening, adherence to the Mediterranean diet, time watching TV or using a computer before bedtime, physical activity, nap during the day) and health related characteristics (subjective general health status, BMI, chronic disease morbidity, anxiety, depression and use of sleep medication) were included in the model; All variables were binary (no, yes); Category “no” forms the reference group.

### CVD and sleep habits

The association of CVD with subjects’ sleep characteristics is shown in Table 4. On weekdays, subjects with CVD used to go to bed earlier (*p*<0.001) and get up earlier (*p*=0.019) compared to subjects without CVD. Time in bed was longer in subjects with CVD (*p*=0.004), while they reported 26 min shorter sleep duration (*p*=0.001) and they had significantly lower sleep efficiency (*p*<0.001) than those without CVD.

**Table 4:** Association of cardiovascular disease (CVD) with sleep characteristics.

		CVD			
		**No**	**Yes**	Difference^*^	**p-value**
Number of subjects		866	91		
Weekday sleep habits					
Bedtime	11:29 (1:05)	11:33 (1:04)	10:50 (1:08)	-43 (7.1)	<0.001
Rise time	6:53 (1:01)	6:55 (1:00)	6:38 (1:04)	-17 (6.7)	0.019
Time in bed	7:24 (1:05)	7:22 (1:03)	7:48 (1:18)	26 (7.1)	0.004
Sleep duration	6:19 (1:11)	6:22 (1:10)	5:56 (1:18)	-26 (7.7)	0.001
Sleep efficiency (%)	86 (12)	87 (11)	77 (11)	-10 (1.2)	<0.001
Weekend sleep habits					
Bedtime	11:55 (1:19)	12:02 (1:16)	10:52 (1:10)	-70 (8.3)	<0.001
Rise time	7:46 (1:32)	7:53 (1:32)	6:41 (1:05)	-72 (9.9)	<0.001
Time in bed	7:50 (1:00)	7:51 (1:00)	7:47 (1:12)	-4 (6.7)	0.534
Sleep duration	6:45 (1:16)	6:50 (1:13)	5:58 (1:18)	-52 (8.2)	<0.001
Sleep efficiency (%)	86 (12)	87 (11)	77 (0.12)	-10 (1.2)	<0.001
Weekly sleep habits					
Total sample					
Time in bed	7:32 (1:00)	7:30 (0:58)	7:47 (1:16)	17 (6.5)	0.047
Sleep duration	6:26 (1:10)	6:29 (1:08)	5:56 (1:18)	-33 (7.6)	<0.001
Sleep efficiency (%)	86 (12)	87 (11)	77 (0.11)	-10 (1.2)	<0.001
Females					
Time in bed	7:36 (0:59)	7:35 (0:58)	7:50 (1:18)	15 (10.2)	0.264
Sleep duration	6:30 (1:10)	6:33 (1:08)	5:50 (1:20)	-43 (11.4)	<0.001
Sleep efficiency (%)	86 (12)	87 (12)	75 (0.13)	-12 (2.0)	<0.001
Males					
Time in bed	7:26 (1:00)	7:24 (0:57)	7:44 (1:16)	20 (8.5)	0.058
Sleep duration	6:22 (1:10)	6:25 (1:08)	6:01 (1:17)	-24 (9.9)	0.015
Sleep efficiency (%)	86 (11)	87 (11)	78 (0.10)	-10 (1.5)	<0.001

Notes:

* Mean difference between subjects with and without CVD, expressed as minutes (bedtime, rise time, time in bed and sleep duration) and percentages (sleep efficiency).

On weekends, although subjects without CVD reported going to bed 29min later (*p*<0.001), getting up 58min later (*p*<0.001) and sleeping 28min more (*p*<0.001) compared to weekdays, the sleep pattern of subjects with CVD remained essentially unchanged (Table 4). In particular, subjects with CVD reported 52min shorter sleep duration (*p*<0.001) and lower sleep efficiency (*p*<0.001) than those without CVD.

In the sequence the weighted weekly values of time in bed and sleep duration were calculated and compared between the two groups (Table 4); it was noted that, although subjects with CVD spent longer time in bed (*p*=0.047), they reported 33min shorter sleep duration (*p*<0.001) and lower sleep efficiency (*p*<0.001) compared to subjects without CVD. All the above relations between CVD and sleep characteristics remained unchanged among men and women (Table 3). In particular, females with CVD used to sleep 43min less than females without CVD (*p*<0.001) and males with CVD used to sleep 24min less than males without CVD (*p*<0.001).

Furthermore, according to the reported sleep duration, participants were categorized into three groups: short (<6h), normal (6-8h) and long (>8h) sleep duration. The association of CVD with sleep duration, which was considered as a categorical variable, is shown in Table 5. CVDs were significantly more frequent (*p*<0.001) in subjects with short (18.7%) compared to those with normal (6.5%) and long (8.8%) sleep duration. The association of CVD with sleep duration exhibited the same pattern in women (*p*=0.010) and men (*p*=0.001). In particular, logistic regression analysis revealed that in subjects with short sleep duration there were more than 3-times higher odds for CVD compared to subjects with normal sleep duration (OR=3.28, *p*<0.001). A 3.07-fold increase in odds of CVD was associated with short sleep duration in females (*p*=0.004) and males (*p*=0.015), respectively.

**Table 5:** Association of sleep duration with cardiovascular disease (CVD) in relation to gender using logistic regression models.

			Model 1		Model 2	
	**CVD** **n (%)**	**p-value**	**cOR (95%CI)**	**p-value**	**aOR (95%CI)**	**p-value**
**Total sample**						
Sleep duration		<0.001				
Short	39 (18.7)		3.28 (2.04-5.27)	<0.001	2.86 (1.71-4.79)	<0.001
Normal	40 (6.5)		Ref.		Ref.	
Long	12 (8.8)		1.38 (0.71-2.71)	0.345	1.41 (0.70-2.82)	0.338
**Females**						
Sleep duration		0.010				
Short	13 (14.1)		3.07 (1.45-6.53)	0.004	2.68 (1.17-6.10)	0.019
Normal	18 (5.1)		Ref.		Ref.	
Long	5 (6.9)		1.39 (0.50-3.88)	0.526	1.05 (0.37-2.99)	0.932
**Males**						
Sleep duration		0.001				
Short	26 (22.2)		3.07 (1.65-5.68)	<0.001	2.57 (1.27-5.20)	0.009
Normal	22 (8.5)		Ref.		Ref.	
Long	7 (10.9)		1.32 (0.54-3.62)	0.548	1.29 (0.50-3.37)	0.592

Notes: cOR = Crude odds ratio; aOR = Adjusted odds ratio; CI = Confidence interval; Model 1 = Crude, unadjusted; Model 2 = Adjusted for sociodemographic characteristics (gender, age, marital status, cultural status, place of residence, education level, working status, financial status), lifestyle habits (smoking status, alcohol consumption, daily coffee consumption, caffeine consumption in the evening, adherence to the Mediterranean diet, time watching TV or using a computer before bedtime, physical activity, nap during the day) and health related characteristics (subjective general health status, BMI, chronic disease morbidity, anxiety, depression and use of sleep medication).

### Independent effect of sleep habits on CVD

Two separate multivariable logistic regression models, controlling for the effect of all subjects’ sociodemographic, lifestyle and health related characteristics, were constructed in order to assess the independent effect of sleep duration on the prevalence of CVD. When sleep duration was entered in the model as a continuous variable, shorter sleep duration remained a statistically significant independent determinant of increased odds for CVD; in particular, shorter sleep duration by one hour was associated with an 29%-increase in the risk for CVD (aOR=1.29, 95%CI=1.05-1.58).

**Figure 1:**
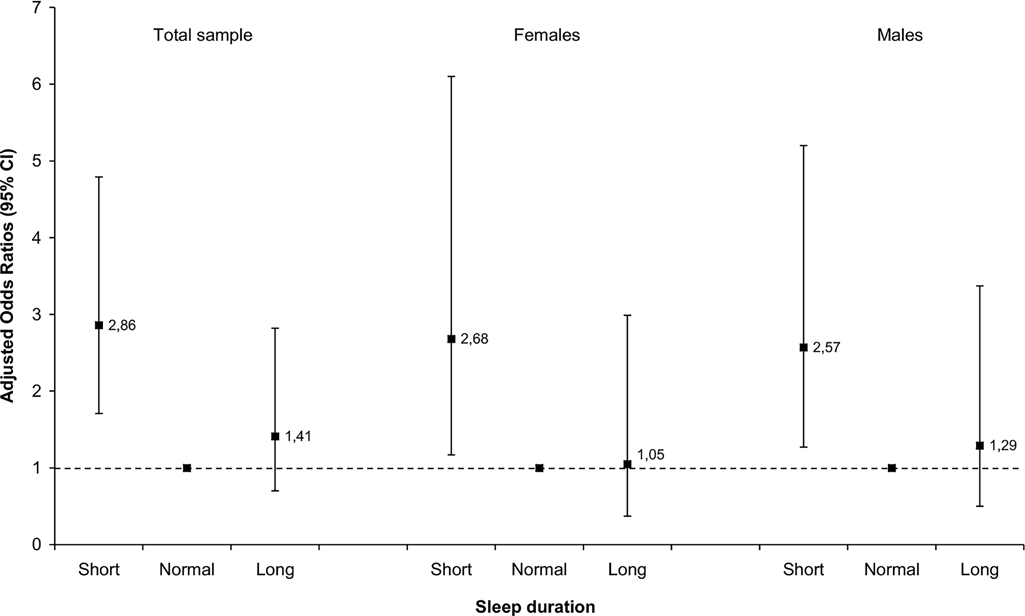
Association of sleep duration with cardiovascular disease (CVD) in relation to gender expressed as adjusted odds ratios and their 95% confidence intervals (CI) obtained from multivariable logistic regression models.

When sleep duration was entered in the multivariable logistic regression model as a categorical variable, the odds for CVD remained higher for the subjects sleeping shorter than 6 hours with adjusted odds ratios of 2.86 (*p*<0.001) in the entire sample, 2.68 (*p*=0.019) in women and 2.57 (*p*=0.009) in men; sleeping longer than 8 hours showed no significant association with CVD (Figure 1).

Moreover, the area under the curve (AUC) showed that sleep duration has a significant ability to discriminate subjects with CVD (AUC=0.663, 95%CI=0.598-0.728, *p*<0.001). The optimal cutoff point of sleep duration of 5:33 hours, which was determined to classify subjects with CVD, yielded high sensitivity of 64.8% and specificity of 77.8%. Sleep duration showed significant discrimination ability in both genders, although its performance was superior among females (females: AUC=0.716, 95%CI=0.615-0.818, *p*<0.001, cutoff ≤5:33 hours, sensitivity=75.0%, specificity=81.5%; males: AUC=0.622, 95%CI=0.538-0.708, *p*=0.003, cutoff ≤5:38 hours, sensitivity=58.2%, specificity=73.2%).

### CVD and sleep disorders

**Figure 2:**
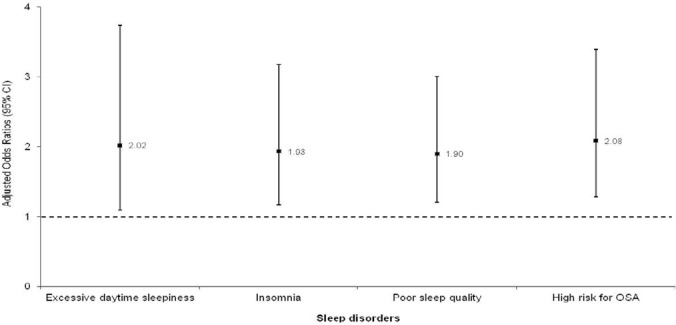
Association of sleep disorders with cardiovascular disease (CVD) expressed as adjusted odds ratios and their 95% confidence intervals (CI) obtained from multivariable logistic regression models.

According to the Greek versions of Epworth sleepiness scale (ESS), Athens insomnia scale (AIS), Pittsburgh sleep quality index (PSQI) and Berlin questionnaire (BQ) the prevalence of daytime sleepiness was 8.7% (83 subjects), insomnia 18.0% (172 subjects), poor sleep quality 38.5% (368 subjects) and high risk of obstructive sleep apnea 36.4% (348 subjects). The internal consistency of all four questionnaires was very high (Cronbach α coefficient ranged from 0.74 to 0.88). The association of CVD with sleep disorders is shown in Table 6. Univariate statistical analysis showed that CVDs were more frequent in subjects with excessive daytime sleepiness (*p*<0.001), insomnia (*p*<0.001), poor sleep quality (*p*<0.001) and higher risk of OSA (*p*<0.001). In multivariable logistic regression analysis controlling for all subjects’ characteristics, the odds of CVD remained higher in subjects with excessive daytime sleepiness (aOR=2.02, *p*=0.026), insomnia (aOR=1.93, *p*=0.010), poor sleep quality (aOR=1.90, *p*=0.006) and higher risk of OSA (aOR=2.08, *p*=0.003) (Figure 2).

**Table 6:** Association of sleep questionnaires and sleep difficulties with cardiovascular disease (CVD) using logistic regression models.

			Model 1		Model 2	
	**CVD** **n (%)**	**p-value**	**cOR (95% CI)**	**p-value**	**aOR (95% CI)**	**p-value**
**Sleep questionnaires**						
ESS		<0.001				
Normal day sleepiness	74 (8.5)		Ref.		Ref.	
Excessive day sleepiness	17 (20.5)		2.79 (1.55-4.99)	<0.001	2.02 (1.09-3.74)	0.026
AIS		<0.001				
Non-insomniac	60 (7.6)		Ref.		Ref.	
Insomniac	31 (18.0)		2.66 (1.66-4.25)	<0.001	1.93 (1.17-3.18)	0.010
PSQI		<0.001				
Good quality	40 (6.8)		Ref.		Ref.	
Bad quality	51 (13.9)		2.21 (1.43-3.42)	<0.001	1.90 (1.20-3.00)	0.006
BQ		<0.001				
Low risk	35 (5.7)		Ref.		Ref.	
High risk	56 (16.1)		3.15 (2.02-4.91)	<0.001	2.08 (1.28-3.39)	0.003
**Sleep difficulties**						
Delay in falling asleep		0.048				
Less than once a week	66 (10.9)		Ref.		Ref.	
At least once a week	25 (7.1)		0.62 (0.38-0.99)	0.938	0.77 (0.47-1.27)	0.303
Inability to stay asleep		<0.001				
Less than once a week	7 (1.9)		Ref.		Ref.	
At least once a week	84 (14.1)		8.34 (3.81-18.24)	<0.001	2.36 (1.25-4.47)	0.008
Waking-up too early		0.008				
Less than once a week	41 (7.4)		Ref.		Ref.	
At least once a week	50 (12.5)		1.80 (1.16-2.78)	0.008	1.64 (1.01-2.68)	0.046

Notes: ESS = Epworth sleepiness scale; AIS = Athens insomnia scale; PSQI = Pittsburgh sleep quality index; BQ = Berlin questionnaire; cOR = Crude Odds Ratio; aOR = Adjusted odds ratio; CI = Confidence interval; Model 1 = Crude, unadjusted; Model 2 = Adjusted for sociodemographic characteristics (gender, age, marital status, cultural status, place of residence, education level, working status, financial status), lifestyle habits (smoking status, alcohol consumption, daily coffee consumption, caffeine consumption in the evening, adherence to the Mediterranean diet, time watching TV or using a computer before bedtime, physical activity, nap during the day) and health related characteristics (subjective general health status, BMI, chronic disease morbidity, anxiety, depression and use of sleep medication).

Among the basic difficulties of sleep patterns, significant increased odds of CVDs were found among subjects who reported difficulties in maintaining sleep (aOR=2.36, *p*=0.008) and early morning awakenings (aOR=1.64, *p*=0.046), but not with difficulties initiating sleep (aOR=0.77, *p*=0.303).

## DISCUSSION

Our research was designed in order to evaluate the possible associations of sleeping habits and disorders with CVD using a representative population-based sample from the rural region of Thrace, in northeastern Greece. The prevalence of CVD was higher among older men, smokers, with sedentary life, low educational and financial status inhabiting the countryside, along with previous studies^25^. Another interesting finding of our study was the changing pattern of CVD distribution in our sample based upon the marital status, with the widowed patients holding the lion’s share, as also concluded in the research of Marzieh et al. (2021)^26^. The overall results revealed a strong correlation of CVD with shorter sleep duration and impaired sleep efficiency, but also high prevalence of excessive daytime sleepiness, insomnia, poor sleep quality and increased risk of obstructive sleep apnea. With regards to insomnia, patients with CVD reported difficulties in maintaining sleep and early morning awakenings, but not difficulties initiating sleep.

In our study, patients with CVD demonstrated significantly shorter sleep duration and lower sleep efficacy compared to individuals without CVD. In particular, mean sleep duration was reduced by 33 min, mean sleep efficiency by 10% and short sleep duration was 3.07-times more frequent in patients with CVD. These results are consistent with the longitudinal study of Covassin et al. (2016)^27^, on a sample of 71,617 participants where CVD presented a 1.39-fold higher prevalence in women reporting ≤5 hours/night compared to those sleeping 8 hours/night. Apart from that, the epidemiologic study of Liu et al. (2013)^28^ with the participation of 54,269 adults pointed out that the prevalence of coronary heart disease was higher in the population with sleep duration less than 6 hours/night. Similarly, according to the meta-analysis by Holliday et al. (2013)^29^, sleep duration of less than 6 hours was significantly associated with increased risk by 30% for type 2 diabetes. Furthermore, Cappuccio et al. (2011)^30^ have proven that the incidence of fatal and non-fatal events of coronary heart disease was almost 1.5 times more frequent in the population with sleep duration of less than 7 hours, acknowledging its prognostic role on cardiovascular disease^5,30^. However, He et al. (2017)^31^ support that long sleep duration is responsible for the impairment of cardiovascular health through possible prothrombotic pathways, thus favoring the risk of stroke.

We have also demonstrated that sleep duration of less than 5:33 hours could be a potential risk factor for CVD, mainly for females, while most literature emphasizes on sleep duration of less than 6 hours as harmful for the cardiovascular burden^32^. Similar results have been shown in the large national cohort by Shankar et al. (2008)^33^ where self-reported sleep less than 5 hours/night by postmenopausal women induced an augmented risk of 25% for coronary heart disease. Kronholm et al. (2011)^34^ also concluded that sleep duration of less than 5 hours/night is an independent risk factor for CVD mortality and morbidity in women.

Concerning sleep quality, our results are in accordance with available studies making use of the aforementioned sleep quality scales^35^. Insomnia and poor sleep quality are associated with increased risk of CVD as also proven respectively by Del Bruto et al. (2008)^36^ and Costa et al. (2017)^37^. Moreover, these results come in line with the conclusion of Maia et al. (2017)^38^ revealing a positive correlation between high risk of obstructive sleep apnea and coronary heart disease mortality in patients following an acute coronary syndrome during a follow-up of almost 3 years. Additionally, the research of Acharya et al. (2020)^39^ has highlighted the importance of obstructive sleep apnea as a risk factor for cardiac arrhythmias and sudden cardiac arrest. Our study has also noted statistical significance for excessive daytime sleepiness as a predisposing factor for cardiovascular disease, a finding corresponding to the findings of the recent study of Xie et al. (2018)^40^.

Another interesting finding of our study was that long sleep duration was not associated with cardiovascular disease. Hamazaki et al. (2011)^41^, Amagai et al. (2004)^42^, Yazdanpanah et al. (2020)^43^, could not also find a relation between long sleep duration and cardiovascular disease. The neutral effect of prolonged sleep on cardiovascular disease is consistent with the research of Domínguez et al. (2019)^44^, where longer sleep duration did not alter subclinical multiterritory atherosclerosis. On the contrary, there are several studies presenting the negative effect of prolonged sleep on cardiovascular health. Ferrie et al. (2007)^45^ concluded that both long and short sleep duration are associated with impairment of cardiovascular mortality. The meta-analysis by Cappuccio et al. (2011)^30^ concluded that long sleep duration was significantly associated with cardiovascular events in a sample of 474,685 participants.

The pathophysiologic mechanisms underlying the connection between sleep disturbances with CVD although not yet totally understood are based mainly on experimental evidence depicting an interaction between brain and heart^46^. The overstimulation of the sympathetic nervous system is suggested to be a major contributor to cardiovascular disease. In particular, subjects with repeated sleep interruptions exhibited higher nocturnal blood pressure accompanied by dampened nocturnal dipping effect^47^ and an enhanced morning rise^48^. A possible explanation resides in the increased cardiac sympathetic drive and cardiovascular over-responsiveness to stress^49^ as estimated by heart rate variability measurements^50^. Another important mechanism impairing the cardiovascular system is the hypothalamic-pituitary-adrenal axis (HPA-axis)^46^. Indeed, this theory is based on increased levels of plasma and urinary norepinephrine and cortisol, leading to stress overload^51^. As a result, data from human population’s link sleep deprivation with aggravation of arterial stiffness^52^, coronary microcirculation^53^ and endothelial function^54^, which may advance atherosclerosis and may cause myocardial damage. Furthermore, the proinflammatory and procoagulant potential of sleep insufficiency is reflected by increased levels of TNF-a, IL-1, IL-6, IL-17, CRP, D-dimers, and fibrinogen^55^. Finally, sleep deficiency affects the metabolic pathways by favoring insulin resistance and weight gain^56^.

Our study overall presents sufficient points of strength which include the following. Firstly, the data of our research derive from a large and representative sample of a regional Greek population, in Thrace. Additionally, the methodology of our sample selection was random, thus reassuring the representation of the general population of this area. The extensive and careful use of diagnostic tools and questionnaires offered acceptable estimates of sleep quality, quantity and cardiovascular disease. The main limitations of our analysis reside in the character of the cross-sectional study, the non-investigation of our subjects’ medication history and the recall bias of self-reported sleep duration instead of techniques such as polysomnography or actigraphy. The use of self-reported questionnaires may affect CVD prevalence as well as overestimate sleep duration and quality especially in the pattern of a rural population accompanied by a low level of education. Despite this restriction, self-report assessments of sleep have been proven to be reliable measures when compared to quantitative sleep assessments with actigraphy^57^.

## CONCLUSION

Our research revealed the increased prevalence of the cardiovascular burden in a regional elder Greek population and the interaction of impaired sleep duration and quality with CVD. Moreover, CVD may induce excessive daytime sleepiness, insomnia, poor sleep quality and increased risk of obstructive sleep apnea. As a result, a balanced sleep duration of 6-8 hours accompanied by a healthy lifestyle is pivotal for the cardiovascular health.

## Appendix

**Appendix 1:** Survey questionnaire that was administered to the participants.

I. Standard sociodemographic characteristics																							
Gender		male									female												
Age (years)		≤40								41-50				51-60				61-70				>70	
Marital status		married					single					divorced						widowed					
Cultural status		Greek Christians										Greek Muslims									expatriated Greeks		
Place of residence		urban							rural														
Education level		low: basic education (≤9 years)										medium: up to high school or technical colleges									high: university		
Employment status		employed										unemployed											
Financial status: mean monthly household income during the past three years		low: ≤1,000 Euros										medium: 1,001-2,000 Euros									high: >2,000 Euros		
**II. Lifestyle and dietary habits**																							
Smoking status		never smoked									ex-smoker								current smoker				
Alcohol consumption		never									occasionally								daily				
Daily coffee consumption (cups/day)		0			1-2						3-4						>4						
Caffeine consumption in the evening (>6 p.m.)		no			yes																		
Adherence to the Mediterranean diet^1^		low			high																		
Time watching TV or using a computer before bedtime		less than 1 hour									1-2 hours								more than 2 hours				
Physical activity^2^		low			high																		
Nap during the day		no			yes																		
**III. Health related characteristics**																							
Subjective general health status		bad (including very bad, bad and fair)													good (including good, very good)								
Body mass index		normal weight (including underweight) (BMI<25)											overweight (25≤ BMI<30)								obese (BMI≥30)		
Chronic disease morbidity^4^		no				yes																	
Depression symptoms^5^		no				yes																	
Use of sleep medication		no				yes																	
Notes: ^1^Adherence to the Mediterranean diet was considered as an indicator of healthy diet, it was assessed by means of the dietary indicator Mediterranean Diet Score (ranged from 0 to 55) and scores >35 were considered as high adherence; ^2^Physical activity was assessed according to self-reported weekly frequency, duration and intensity of regular exercise (running, cycling, swimming, football, basketball, tennis, volleyball) and walking. Moderate exercise for at least 3 days per week or walking for at least 5 days per week for at least 30 min per day was classified as high physical activity; ^3^Body mass index (BMI; weight/height^2^ in kg/m^2^) was calculated and categorized according to the World Health Organization (WHO) criteria; ^4^Chronic disease morbidity was defined as the self-reported preexisting health problems, such as: hypercholesterolemia, hypertension, rheumatic disease, allergy, gastrointestinal disease, cardiac disease, diabetes, pulmonary disease, cancer, and neurologic disease; ^5^Depression symptoms were assessed using the Greek version of the Beck depression inventory (BDI) (a 21-item self-reporting scale; cutoff point: 13).																							
**Assessment of sleep habits**																							
			On weekdays											On weekends									
At what time do you normally go to bed?																							
At what time do you normally get up?																							
On average, how many hours do you sleep per day?																							
**Standardized scales in Greek version that were used for the assessment of sleep disturbances**																							
**Epworth Sleepiness Scale (ESS)^1^**	Normal daytime sleepiness			Excessive daytime sleepiness				**Pittsburgh sleep quality index (PSQI) ^3^**								Good sleeper				Poor sleeper			
**Athens Insomnia Scale (AIS) ^2^**	Non-insomniac			Insomniac				**Berlin questionnaire (BQ) ^4^**								Low risk				High risk			
Notes: ^1^ ESS is a widespread tool for evaluating subjective excessive daytime sleepiness. It includes 8 questions pertaining to day-to-day activities. Subjects are asked to rate the chance of dozing off or falling asleep in 8 different situations. The score of each question ranges from 0 to 3, and their sum is the final score of the questionnaire. Higher scores indicate a higher average sleep propensity. Based on the results, patients were classified into 2 subgroups: 0-10 normal daytime sleepiness and >10 excessive daytime sleepiness; ^2^AIS is a common self-assessment measure of insomnia-related symptoms designed for quantifying sleep difficulty based on the ICD-10 criteria. It consists of eight items: the first five pertain to sleep induction, awakenings during the night, final awakening, total sleep duration, and sleep quality; while the last three refer to well-being, functioning capacity, and sleepiness during the day. A score of >5 on the AIS was used to establish the diagnosis of insomnia; ^3^PSQI is a standard self-assessment of sleep quality over the last month. It consists of 19 questions grouped into 7 subcategories. Specifically, the seven distinct clinical subclasses of sleep difficulties are as follows: (1) subjective sleep quality (one question), (2) sleep latency (two questions), (3) sleep duration (one question), (4) sleep efficiency (three questions), (5) sleep disorders (nine questions), (6) use of sleep medication (one question), (7) daytime dysfunction (two questions). These distinct subcategories are summed and produce an overall result, with a normal value of ≤5. Thus, subjects are divided into good sleepers (PSQI≤5) and poor sleepers (PSQI>5); ^4^The Berlin questionnaire is one of the most common tools for identifying patients with an increased risk of OSA. It consists of 10 questions grouped into 3 categories: (1) snoring severity (questions 1-5), (2) daytime sleepiness or fatigue (questions 6-9), (3) history of arterial hypertension or obesity (question 10). The questions also include information about sex, age, height, and weight. Individuals are at high risk of OSA if they are positively rated in at least 2 categories, and if they are scored positively in one or none of the categories, the risk is low.																							
